# Predicting Gleason sum upgrading from biopsy to radical prostatectomy pathology: a new nomogram and its internal validation

**DOI:** 10.1186/s12894-020-00773-5

**Published:** 2021-01-06

**Authors:** Xiaochuan Wang, Yu Zhang, Fengbo Zhang, Zhengguo Ji, Peiqian Yang, Ye Tian

**Affiliations:** Department of Urology, Capital Medical University Affiliated Beijing Friendship Hospital, No. 95, Yongan Road, Xicheng District, Beijing, People’s Republic of China

**Keywords:** Prostatic neoplasms, Neoplasm grading, Biopsy, needle, Prostatectomy, Nomograms

## Abstract

**Background:**

To explore the rate of Gleason sum upgrading (GSU) from biopsy to radical prostatectomy pathology and to develop a nomogram for predicting the probability of GSU in a Chinese cohort.

**Methods:**

We retrospectively reviewed our prospectively maintained prostate cancer (PCa) database from October 2012 to April 2020. 198 patients who met the criteria were enrolled. Multivariable logistic regression analysis was performed to determine the predictors. Nomogram was constructed based on independent predictors. The receiver operating curve was undertaken to estimate the discrimination. Calibration curve was used to assess the concordance between predictive probabilities and true risks.

**Results:**

The rate of GSU was 41.4%, whilst GS concordance rate was 44.4%. The independent predictors are prostate specific antigen (PSA), greatest percentage of cancer (GPC), clinical T-stage and Prostate Imaging Reporting and Data System (PI-RADS) score. Our model showed good discrimination (AUC of 0.735). Our model was validated internally with good calibration with bias-corrected C-index of 0.726.

**Conclusions:**

Utilization of basic clinical variables (PSA and T-stage) combined with imaging variable (PI-RADS) and pathological variable (GPC) could improve performance in predicting actual probabilities of GSU in the 24-core biopsy scheme. Our nomogram could help to assess the true risk and make optimal treatment decisions for PCa patients.

## Background

Gleason score is a critical factor for both risk stratification and disease management in prostate cancer (PCa). However, it was reported that the concordance of Gleason score between initial biopsy and final radical prostatectomy (RP) pathology was barely satisfactory [1]. Gleason sum upgrading (GSU) may affect assessment of the true PCa risk and treatment options for PCa patients. For instance, active surveillance is recommended for patients with Gleason 6 or 3 + 4 but not appropriate for patients with Gleason 4 + 3 or above [2]. Patients with organ-confined tumors of Gleason 8 or above should underwent RP followed by lymph node dissection and other ancillary therapies in case of PSA failure [3]. Similarly, external beam radiation therapy is recommended to be combined with androgen deprivation therapy in patients with Gleason 4 + 3 or above [4]. It has been demonstrated in large-scale studies that patients with GSU were significantly associated with biochemical recurrence, distant metastasis and death from PCa [5, 6].

To address this matter, our study aimed to examine the rate of GSU between biopsy and final pathology and determine independent predictors for GSU. Moreover, our study developed a nomogram including clinical, imaging and pathologic variables based on Chinese population. We therefore expect to help reassess the risk after biopsy and select optimal treatment modalities for PCa patients after a comprehensive evaluation.

## Methods

### Data acquisition and patient selection

From our prospectively maintained institutional review board-approved prostate biopsy database, we identified 2039 consecutive men who underwent biopsy at our institution (1st October 2012 to 30th April 2020). 212 patients who underwent RP at our institution met the inclusion criteria. The exclusion criteria were as follows: (1) patients treated with neoadjuvant therapy before RP; (2) patients with missing data. Finally, 198 eligible patients entered into our study cohort. Two researchers independently browsed the database, extracted the data and cross-checked. This study did not contain any human participants or animals and it received ethics approval from Capital Medical University affiliated Beijing Friendship Hospital Ethics Committee for database access (2019-P2-081-01).

### Clinical evaluation

Prostate volume (PV) was calculated using anterior–posterior diameter, superior-inferior diameter and left–right diameter which were obtained from multiparametric magnetic resonance imaging (mpMRI). Prostate specific antigen (PSA) was measured before digital rectal examination (DRE) and biopsy. Clinical T-stage was assigned according to the 2017 TNM system which relied on findings of biopsy, DRE and mpMRI.

As recommended [7], the prostate mpMRI was performed before biopsy at a 3-T (T) system. MpMRI protocol consisted of T2-weighted imaging (T2WI), diffusion-weighted imaging (DWI) with apparent diffusion coefficient map (ADC), and dynamic contrast-enhanced (DCE) sequences and calculated b value of 1000 or above. Urological radiologists who were experienced with PI-RADS score and were blinded to pathology as well as clinical data evaluated all the images and performed scoring.

### Biopsy and pathological evaluation

Patients received 18-gauge transperineal needle biopsy under general anesthesia in a dorsal lithotomy position. The biopsy was performed under the guidance of transrectal ultrasonography (TRUS) utilizing an 24-core extended scheme. There were twenty needles in the peripheral zone (PZ) from apex and mid to posterior base and four needles in the transitional zone (TZ). Each core was submitted in a separate container. 24-core systematic biopsy was a standard practice across our center performed by experienced urologists. Gleason sum was assigned core by core by a specialized team of urogenital pathologists using global Gleason score. All RP specimens were examined for prostatectomy Gleason sum by the same team. Hence, non-uniform interpretation of reports between pathologists and clinicians could be avoided [8]. Tertiary patterns were included in biopsy, but not included in prostatectomy specimens. GSU was defined as any Gleason sum upgrading from biopsy to RP. GPC (greatest percentage of cancer in a single core) and fraction of positive cores (FPC) were applied to measure tissue tumor extent (TTE) in biopsy cores [9].

### Statistical analysis

The patients were divided into two groups as those with GSU and those without GSU. Age, metabolic status, body mass index (BMI), interval from biopsy to RP, PV, PSA, PSA density (PSAD), clinical T-stage, PI-RADS score and biopsy specimen features were analyzed in all patients. Normality of distribution of the variables was checked using the Shapiro–Wilk tests and P–P plots. Normally distributed numerical variables were analyzed by the student *t* tests. Mann–Whitney U tests were applied to determine the significance of nonnormally distributed numerical variables. Chi-square tests were used for categorical variables. Univariable regression analysis was performed followed by the multivariable analysis. Variables that were found statistically significant in univariable analysis entered the multivariable analysis in a forward stepwise selection (probability of 0.05). The nomogram was constructed with validated independent predictors. The performance of the prediction model was evaluated from the aspect of discrimination and calibration. Discrimination was measured using the receiver operating curve (ROC) with the area under the curve (AUC) value. Calibration was assessed by visually inspecting the plots of predicted probability and actual probability. Bias-corrected C-index was also calculated to be compared with original C-index (AUC value). Internal validation was performed by bootstrap resampling (n = 1000) to evaluate the accuracy estimates and to reduce overfit bias. Tests were 2 sided and *P* < 0.05 was the threshold for statistical significance. Statistical tests were performed using computer software of SPSS version 24.0 and R version 4.0.2.

## Results

A statistical significance was met when it came to variables: cT-stage, PI-RADS score, PSA and GPC (all *P* < 0.05). Patients in GSU subgroup had a higher ratio for T_2b-2c_ cancers (66.3%) and PI-RADS score of 4–5 (89.9%), higher PSA level (15.7 ng/ml) and higher GPC (90.0%). Other demographic details were shown in Table 1.

**Table 1:** Demographics of patients underwent biopsy followed by prostatectomy in total and subgroups

Variables	Total (n = 198)	GSU (n = 89)	Non-GSU (n = 109)	*P* value
Age at biopsy (year), M(IQR)	67.0(63.0–71.0)	67.0 (62.0–71.0)	67.0 (63.0–71.0)	0.496^a^
MS (n, %)	–	–	–	0.150
Absence	156(78.8)	66 (74.2)	90 (82.6)	–
Presence	42(21.2)	23 (25.8)	19 (17.4)	–
BMI, M(IQR) (kg/m^2^)	24.7(22.9–26.9)	25.0(23.1–27.2)	24.6(22.3–26.7)	0.345^a^
Interval from biopsy to RP, M(IQR) (d)	21.0(14.0–34.3)	21.0(14.0–31.5)	21.0(14.0–35.0)	0.771^b^
PV, M(IQR) (ml)	39.0(28.1–53.0)	38.8(28.7–52.8)	39.2(23.9–53.4)	0.801^b^
Pre-biopsy PSA level, M(IQR) (ng/ml)	14.2(8.6–28.7)	15.7(9.1–35.9)	13.1(7.8–23.6)	0.032^b^
Pre-biopsy PSAD, M(IQR) (ng/ml^2^)	0.37(0.21–0.79)	0.46(0.23–1.01)	0.34(0.20–0.74)	0.061^b^
Clinical T-stage (n, %)	–	–	–	0.002
T_1c-2a_	63(31.8)	18 (20.2)	45 (41.3)	0.001^c^
T_2b-2c_	105(53.0)	59 (66.3)	46 (42.2)	0.117^d^
T_3a-3b_	30(15.2)	12(13.5)	18 (16.5)	0.270^e^
PI-RADS score (n, %)	–	–	–	< 0.001
2–3	51(25.8)	9 (10.1)	42 (38.5)	–
4–5	147(74.2)	80 (89.9)	67 (61.5)	–
Cores obtained (n, %)	–	–	–	0.480
12–23	16(8.1)	9(10.1)	7(6.4)	–
24	165(83.3)	74(83.1)	91(83.5)	–
25–30	17(8.6)	6(6.7)	11(10.1)	–
FPC, M(IQR) (%)	25.0(13.0–42.0)	25.0(12.5–48.0)	25.0(13.0–42.0)	0.906^b^
GPC, M(IQR) (%)	70.0(30.0–90.0)	90.0(50.0–90.0)	50.0(30.0–90.0)	0.001^b^
Experience in biopsy (n, %)	–	–	–	0.886
Senior	79(39.9)	36(40.4)	43 (39.4)	–
Junior	119(60.1)	53 (59.6)	66 (60.6)	–

*GSU* Gleason sum upgrade, *M* median, *IQR* interquartile range, *MS* metabolic syndrome, *RP* radical prostatectomy, *BMI* body mass index, *PV* prostate volume, *PSA* prostate specific antigen, *PSAD* PSA density, *PI-RADS* prostate Imaging Reporting and Data System, *FPC* fraction of positive cores, *GPC* the greatest percentage of cancer

^a^Analyzed by the student *t* test

^b^Analyzed by the Mann–Whitney U test

^c^Comparison between T_1c-2a_ and T_2b-2c_

^d^Comparison between T_2b-2c_ and T_3a-3b_

^e^Comparison between T_1c-2a_ and T_3a-3b_

**Fig. 1:**
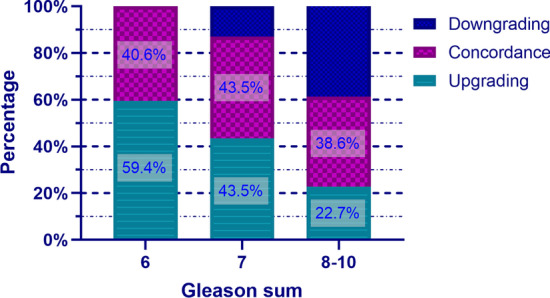
Stacked bar chart of the distribution and magnitude of Gleason sum change from biopsy to prostatectomy pathology in three cohorts of men with different Gleason sum

We found that concordance rate between initial biopsy pathology and final RP specimens was 41.4% (82/198), whilst GSU was 44.4% (88/198). Downgrading was found in 14.1% (28/198) of patients (Table 2). Our study further divided the entire cohort into three groups according to the biopsy Gleason sum (6, 7, ≥ 8). The percentage of GSU was highest in patients with Gleason sum 6, while the concordance rate was highest in patients with Gleason sum 7. The percentage of downgrading was highest in patients with Gleason sum 8–10 (Fig. 1).

**Table 2:** Distribution in Gleason scores between the biopsy cores and RP specimens

Biopsy Gleason sum	RP Gleason sum						
	6	3 + 4	4 + 3	8	9	10	Total
6	28	28	4	6	3		69
3 + 4	3	24	9	13	4		53
4 + 3	1	7	13	10	1		32
8		2	9	7	9		27
9		1	2		9	1	13
10				1	2	1	4
Total	32	62	37	37	28	2	198

**Fig. 2:**
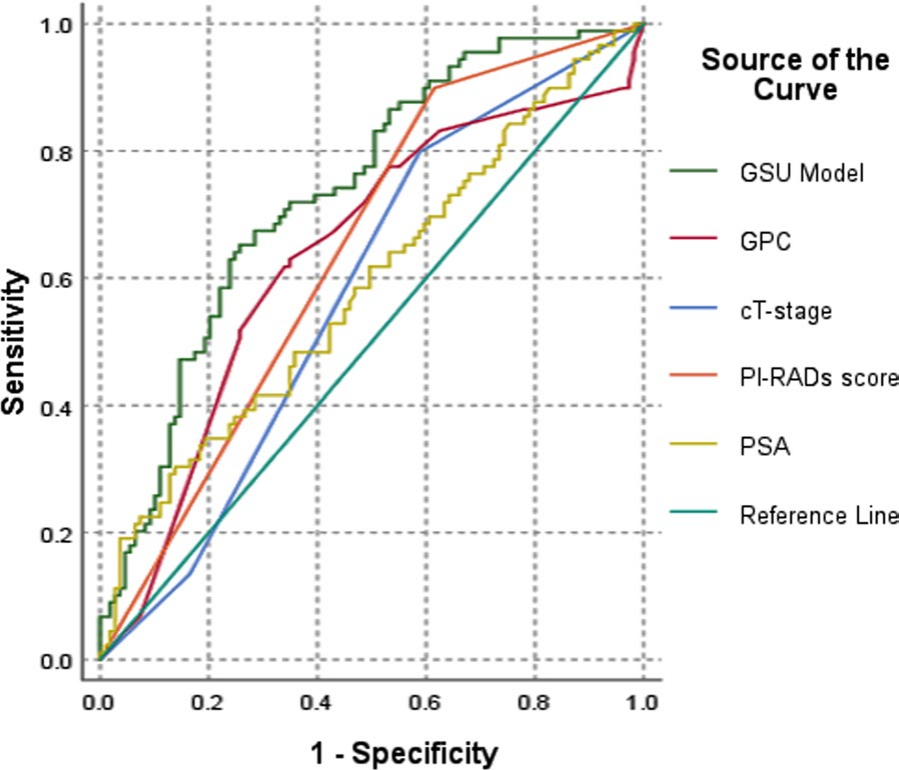
ROC of Gleason sum upgrading model and independent predictors. *GSU* Gleason sum upgrading, *GPC* the greatest percentage of cancer, *PSA* prostate specific antigen, *PI-RADS* Prostate Imaging Reporting and Data System

**Fig. 3:**
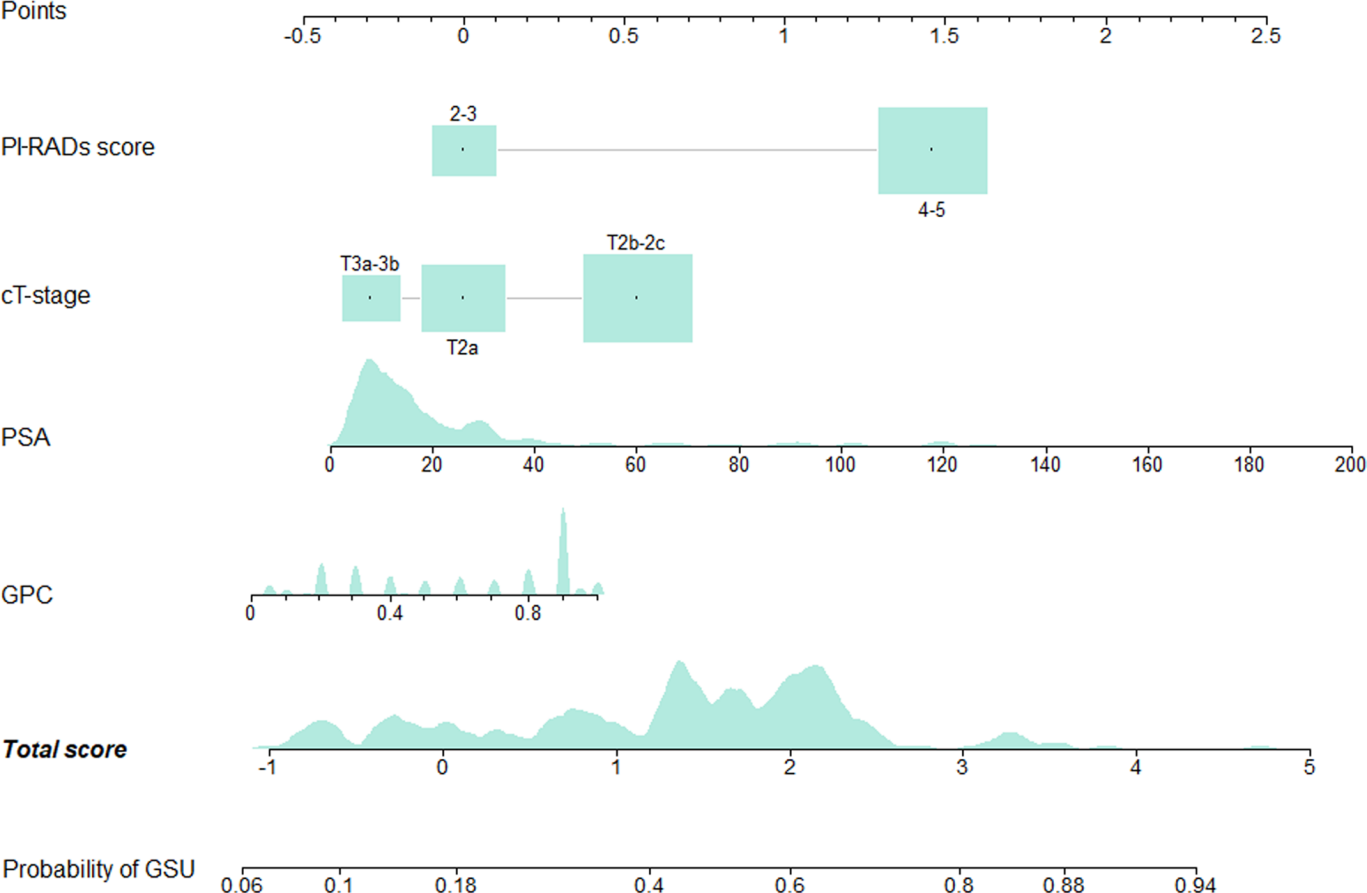
Nomogram of predicting the probability of Gleason sum upgrading. To obtain the predicted probability of GSU, locate each variable of one patient at corresponding axis. Draw a vertical line to the “Points” axis to determine how many points are attributed for each variables. Sum the points of all variables. Locate the sum point on the “Total Points” line to determine the individual probability of GSU on the “Probability of GSU” line. *GSU* Gleason sum upgrading, *GPC* the greatest percentage of cancer, *PSA* prostate specific antigen, *PI-RADS* Prostate Imaging Reporting and Data System

Univariate analysis revealed that PSA, PSAD, GPC, cT-stage and PI-RADS score were the predictors of GSU (all *P* < 0.05). Multivariate analysis confirmed that PSA, GPC, cT-stage and PI-RADS score were independent predictors of GSU (all *P *< 0.05) (Table 3). Additionally, we managed to find whether there were predictors for downgrading and consequently no significant factor was found in regression analysis. The nomogram was constructed based on four predictors of multivariable analysis. The AUC of GPC, PSA, PI-RADS score and cT-stage were 0.636, 0.589, 0.642 and 0.579 respectively. The AUC of GSU model was 0.735, which showed good performance of discrimination (Figs. 2, 3).

**Table 3:** Predictors of Gleason sum upgrading from biopsy to RP

Variables	Univariable analysis		Multivariable analysis	
	OR (95% CI)	*P* value	OR (95% CI)	*P* value
Age at biopsy	0.98(0.94–1.03)	0.494	–	–
MS, presence vs absence	1.65 (0.83–3.28)	0.152	–	–
BMI	1.05 (0.95–1.15)	0.343	–	–
Interval from biopsy to RP	1.00(0.99–1.00)	0.253	–	–
PV	1.00(0.99–1.01)	0.713	–	–
Pre-biopsy PSA level	1.01(1.00–1.02)	0.009	1.02(1.00–1.03)	0.007
Pre-biopsy PSAD	1.42(1.01–2.01)	0.044	0.41(0.14–1.12)	0.522
Clinical T-stage	–	–	–	–
T1c-2a	1.00(Reference)	-	1.00(Reference)	–
T2b-2c	3.21(1.64–6.26)	0.001	4.13(1.82–7.24)	0.042
T3a-3b	1.67(0.67–4.15)	0.272	2.39(0.86–4.85)	0.122
PI-RADS score 4–5 versus 2–3	5.57(2.53–12.27)	< 0.001	4.81(2.07–11.18)	< 0.001
Cores obtained	–	–	–	–
12–23	1.00(Reference)	–	–	–
24	0.63(0.23–1.78)	0.385	–	–
25–30	0.42(0.10–1.72)	0.231	–	–
FPC	1.24(0.37–4.14)	0.731	–	–
GPC	5.34(1.98–14.39)	0.001	3.09(1.05–9.07)	0.040
Experience in biopsy, senior versus junior	1.04(0.59–1.85)	0.886	–	–

*OR* odds ratio, *CI* confidence interval, *MS* metabolic syndrome, *RP* radical prostatectomy, *BMI* body mass index, *PV* prostate volume, *PSA* prostate specific antigen, *PSAD* PSA density, *PI-RADS* Prostate Imaging Reporting and Data System, *FPC* fraction of positive cores, *GPC* the greatest percentage of cancer

**Fig. 4:**
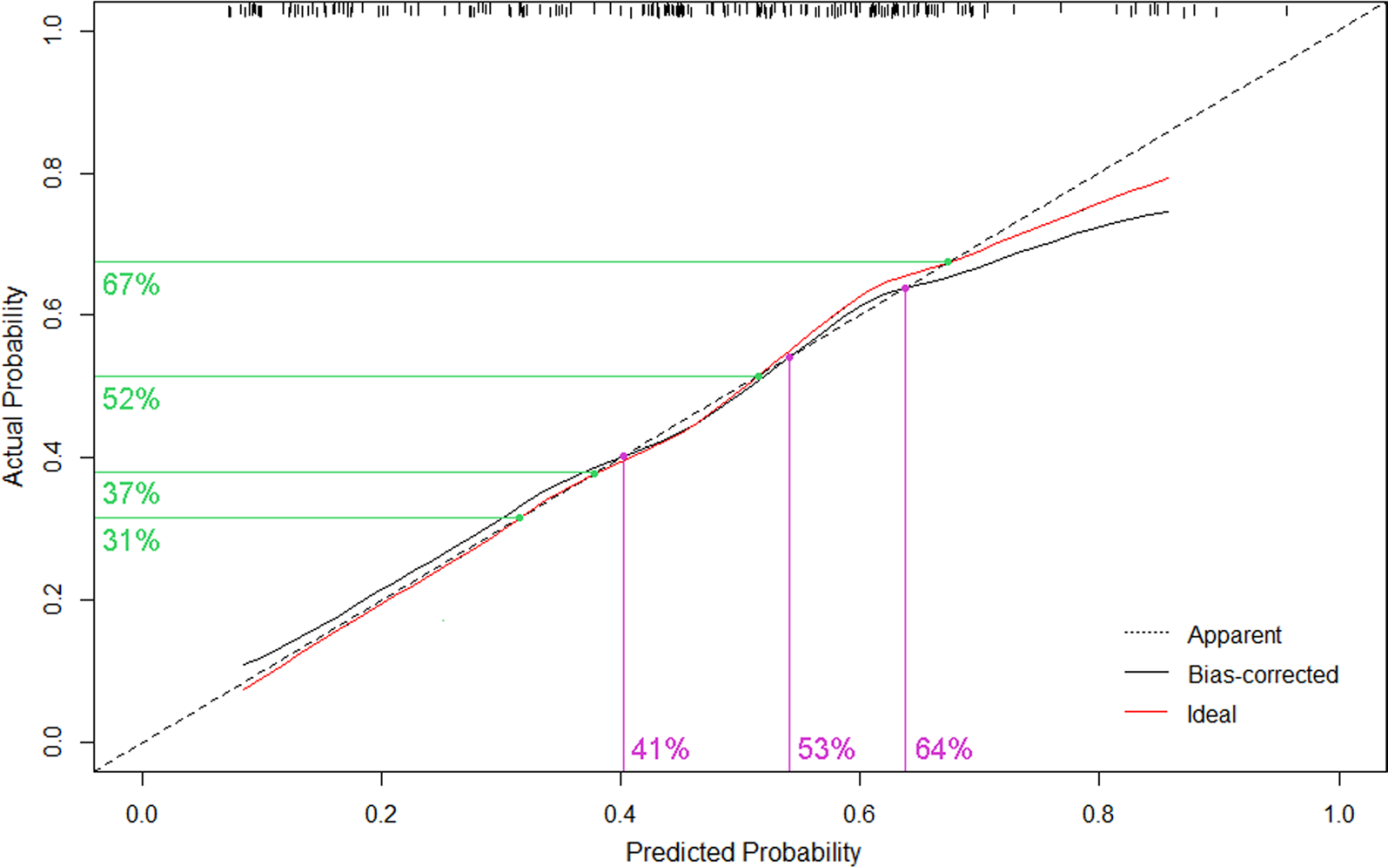
Calibration curve of the Gleason sum upgrading model. The nomogram-predicted probabilities are comparable to the actual probabilities of GSU. Perfect prediction would correspond to a slope of 1 (diagonal 45° broken line). The solid red line indicates the original predictive ability. The solid black line indicates the bootstrap-corrected model performance

Our internally validated calibration plot demonstrated virtually ideal predictions that the rate of predicted probabilities closely paralleled the observed rate (nearly corresponded to the 45° line) with bias-corrected C-index of 0.726 (Fig. 4). Our model predicting GSU might underestimate the risk at the probability range of 53–64% and below 41%. The overestimate risk was at the range of 42–52% and above 64%.

## Discussion

A systematic review including 14,839 patients reported that concordance rate was 63%, while overall upgrading was found in 30% [1]. Based on British Association of Urological Surgeons (BAUS) Radical Prostatectomy Registry database, a study of 17,598 patients [10] demonstrated that concordance rate was 58.9%, whilst upgrading rate was 25.5%. Our study showed that the concordance rate was 41.4%, while GSU was 44.4%. Concordance rates and upgrading rates vary from study to study which might be attributed to reasons as follows: different demographic characteristics of study cohorts, sampling error of biopsy approach, different biopsy techniques, variability of pathologic assignment and non-uniform interpretation of pathology reports.

PSA is a widely used indicator for both risk stratification and prognosis evaluation, and it also plays a role in predicting Gleason sum upgrading [11–19]. High level of serum PSA was partially attributed to large prostate glands in elder patients. However, Davis et al. [18] found that PSA was still an independent predictor after adjusting for PV. In the current study, we found that PSA was also predictive and increasing PSA level was strongly correlated with GSU. For every increasing PSA unit, there was a 2% increased risk of upgrading. Hence, urologists should raise concern about the cumulative effect in patients with high level of PSA.

Clinical T-stage is another important preoperative indicator as for guiding treatment decisions and evaluating prognosis, and it was also found to be predictive of upgrading in several studies [11, 12, 15–17]. Advanced T-stage is in general correlated with large tumor volume and sampling error trends to appear when biopsies are performed in patients with large tumor focus. However, T_2_ tumors were associated with more upgrading compared to T_3_ tumors in the study of Chun et al. [12], the finding of which was consistent with the current study. PCa occurs mainly in the PZ which is compressed by hyperplastic TZ tissue in elder patients. Under this circumstance, small tumor volume and tumor extracapsular extension might coexist. Additionally, diagnostic accuracy of predicting RP Gleason sum varied in different prostate zones which might give another explanation to this phenomenon [20]. However, the correlation between GSU and clinical T-stage needs further investigations.

GPC which was commonly used by pathologists to determine TTE was reported to be correlated with upgrading [13, 18, 19, 21]. In the current study, this pathologic variable was an independent predictor of GSU. High maximum percentage of cancer reflects larger tumor volume which might increase sampling error. Under this circumstance, high-grade masses in large tumor foci would be less detected. Even if small GPC provides pathologists with inadequate information which could increase the possibilities of incorrect scoring, it seems that impacts on the assignment of primary and secondary patterns are slight.

PI-RADS score was validated to have good performance for both PCa detection and foci localization [22]. The utilization of mpMRI was confirmed the value of differentiating clinically significant PCa from indolent tumors [23]. We found that PI-RADS score was also an independent predictor of GSU, which is consistent with the findings of Song et al. [24]. Gleason sum were more likely to be upgraded among patients with PI-RADS 4–5 (54.4% compared with 17.6% for PI-RADS 2–3). High-grade patterns with MRI manifestation of PI-RADS 4–5 which are not identified in biopsy cores result in GSU. Gondo et al. [25] found that Gleason sum downgrading was associated with negative MRI findings, which demonstrated that mpMRI might have favorable diagnostic performance in predicting Gleason sum change.

We successfully developed and validated a model predicting Gleason sum upgrading from biopsy to RP utilizing clinical variables (PSA and T-stage) combined with imaging variable (PI-RADS) and pathological variable (GPC). Our model is 73.5% accurate and its predictions closely approximate the observed rate. Our study was not the first one to construct models or nomograms. Chun et al. [12] built a model including PSA, clinical stage and biopsy Gleason score and yielded accuracy of 0.804 based on a large scale of population. The number of biopsy cores ranged from 6 to 12 in their study. However, excluding the number of cores from multivariable analysis would affect the stability of their model. Moussa et al. [16] incorporated manifold variables especially pathologic parameters into their model. However, including statistically insignificant variables leaded to their model becoming unstable. Therefore, their model yielded a C-index of 0.68. Both Kulkarni et al. [14] and Capitanio et al. [26] have built models for patients with Gleason sum 6, which yielded accuracy of 0.71 and 0.66 respectively. These two models fit low-risk population well, however, which could not represent the real situation of overall GSU. Both Wang et al. [17] and He et al. [27] developed models based on Chinese population. The model of Wang et al. [17] included only PSA, clinical stage and biopsy Gleason score which showed favorable statistical performance with C-index of 0.795. Wang et al. also externally validated Chun’s model in the set of Chinese population and the results showed poor concordance between the predicted and observed probabilities. What Wang et al. have found demonstrated that new models need to be constructed to fit specific population and should be further validated with large patient cohorts. He et al. [27] built models predicting upgrading for both overall patients and patients with GS = 6. Two models including variables such as BMI and FPC showed good performance and yielded the AUC of 0.753 and 0.727 respectively. Our model incorporated not only basic clinical features such as PSA and clinical stage, but also pathologic and imaging parameters, which is more suitable for clinical practice. Including independent predictors leaded to our model becoming more stable and the predicted probabilities closely approximated to the actual risk in calibration plot. When probabilities ranged from 53 to 64% and below 41%, underestimation of the risk occurred, while overestimation of risk ranged from 42 to 52% and above 64%.

According to our present study, several clinical implications might be taken into consideration. Patients who have low-risk PCa but high probabilities of GSU could consider curative therapies instead of active surveillance in case of delayed treatment. For these patients who are under active surveillance could adhere to more active follow-up policies. On the contrary, patients with low probabilities of GSU who are unwilling to receive aggressive treatment or have contraindications of operation or radiotherapy are more confidence to undergo the active surveillance. According to the probabilities of GSU, extended periods of close clinical follow-up could be warranted. Moreover, patients might benefit from resection of neurovascular bundle or lymph node dissection who are at high risk of GSU. Similarly, the hormonal therapy as ancillary treatment to radiotherapy might be considered in patients with high probabilities of GSU. These clinical recommendations might give urologists more confidence in clinical decision-making. New model might provide urologists not only precise and comprehensive assessment of PCa risk but also personalized and optimal treatment options for PCa patients. However, there is still far to go before robust evidence emerges.

There are limitations of the present study. First of all, information from database included potential inaccuracy due to the retrospective nature. Secondly, our study was lack of large population because of our strict indications to perform RP and monocentric design. Thus, we need further prospective large-scale studies with selective patients for validation. Thirdly, external validation for our model would be furtherly necessitated, even if our model served as a statistically well-performed tool. Fourthly, only patients undergone RP were selected into cohort which might not represent the reality. Finally, the accuracy of our model could potentially be improved by integrating findings of genetic and molecular biomarker analysis.

## Conclusions

Utilization of basic clinical variables (PSA and T-stage) combined with imaging variable (PI-RADS) and pathological variable (GPC) could improve performance in predicting actual probabilities of GSU in the 24-core biopsy scheme. Our nomogram could help to assess the true risk and make optimal treatment decisions for PCa patients. However, our nomogram needs to be tested for the performance in an external dataset or other study cohorts.

## Data Availability

The datasets used and/or analysed during the current study are available from the corresponding author on reasonable request.

## Abbreviations

GSU: Gleason sum upgrading
PCa: Prostate cancer
RP: Radical prostatectomy
MS: Metabolic syndrome
BMI: Body mass index
PV: Prostate volume
PSA: Prostate specific antigen
PSAD: PSA density
PI-RADS: Prostate Imaging Reporting and Data System
mpMRI: Multiparametric magnetic resonance imaging
GSU: Gleason sum upgrade
GPC: Greatest percentage of cancer
FPC: Fraction of positive cores
TTE: Tissue tumor extent
ROC: Receiver operating curve
AUC: Area under the curve
